# Factor structure and psychometric properties of an Arabic version of the Internet Gaming Disorder Scale, short form (IGDS-SF9)

**DOI:** 10.3389/fpubh.2023.1231550

**Published:** 2023-11-08

**Authors:** Mogeda El Sayed El Keshky, Tmader Alballa

**Affiliations:** ^1^Department of Psychology, Faculty of Arts and Humanities, King Abdulaziz University, Jeddah, Saudi Arabia; ^2^Department of Mathematics, College of Sciences, Princess Nourah bint Abdulrahman University, Riyadh, Saudi Arabia

**Keywords:** internet gaming disorder, Internet Gaming Disorder Scale (IGDS-SF9), psychometric properties, scale validation, factor structure, Saudi Arabia

## Abstract

**Background:**

In its most recent edition of the Diagnostic and Statistical Manual of Mental Disorders (DSM), the American Psychological Association recognized that internet gaming disorder is a psychological condition and provides nine criteria for its diagnosis, and the Internet Gaming Disorder Scale, short form (IGDS-SF9) was developed to assess individuals.

**Aim:**

To develop and investigate the psychometric properties of an Arabic version of IGDS-SF9.

**Method:**

A five-stage translation and verification process produced an Arabic version of IGDS-SF9, and a study was conducted using a sample of 410 respondents (45.6% females, mean age = 17.6, SD = 1.56) to assess its psychometric properties. Respondents completed the Arabic version, the Smartphone Addiction Scale, the Smartphone Application-Based Addiction Scale, the Hospital Anxiety and Depression Scale, as well as a demographic survey. Exploratory and confirmatory factor analyses, measurement invariance, item response theory, convergent, concurrent, criterion validity, and internal consistency reliability and test–retest reliability were employed to validate the scale.

**Results:**

The exploratory factor analysis suggested the scale’s unidimensional structure; the confirmatory factor analysis exhibited good model fit and factor loadings. Measurement invariance, and concurrent, convergent, and criterion validity were established, resulting in good internal consistency and test–retest reliability for the scale.

**Conclusion:**

The Arabic version of IGDS-SF9 is a valid and reliable measure for research and diagnosis in Arabic-speaking countries.

## 1. Introduction

Online videogames are a part of life for many people worldwide. The U.S. Department of Commerce estimated 2.7 billion gamers globally as of 2020 (1). Spending time playing games on the internet can be entertaining, however, evidence suggests that videogames are one of the most addicting technologies (2). Excessive time playing videogames may result in, or be an indication of, a behavioral addiction known as internet gaming disorder (IGD). The rates of IGD are likely range from 5 and 8% in North America (3, 4), 0.2 and 12% in Europe (5, 6), and 8 and 46% in Asia (7, 8). These differences may result from differences in assessments and conceptualizations of this disorder (9).

Neuroimaging studies have shown similarities between IGD and other types of addiction, particularly those that are substance-related (10). Numerous negative psychosocial consequences have been associated with IGD, including attention deficit hyperactivity disorder (11), aggressive behaviors (12, 13) and violent tendencies (14), poor impulse control (15), low academic performance (16), loneliness (17), anxiety and depression (18), reduced life satisfaction (19), reduced psychological well-being (20), maladaptive coping behaviors (21), as well as delinquency and antisocial behaviors (22).

The APA *Diagnostic and Statistical Manual of Mental Disorders, 5th edition (DSM-5)* recognizes IGD as a disorder in the section recommending conditions requiring further research (23). The DSM-5 proposes nine criteria for diagnosing IGD: preoccupation with gaming; withdrawal symptoms like sadness, anxiety, and irritability when it is not possible to play; tolerance, that is, wanting to spend more time gaming to quench the desire to play; being unable to lessen or quit playing; lost enthusiasm for previously pleasurable activities; continued gaming in spite of other difficulties; deception of others about time involved in gaming; assuaging negative moods by gaming; and gaming endangering or causing the loss of a job or relationship. IGD can be diagnosed when a minimum of five of these criteria persist for 12 months (23) and is specified as the “persistent and recurrent use of the internet to engage in games, often with other players, leading to impairment or clinically significant distress” [(23), p. 795]. This definition relates to online gaming, but the IGD criteria also allow for non-internet videogames (23).

Consequently, to facilitate research and diagnosis measures to assess IGD, such as the Internet Disorder Scale, Short Form (IGDS-SF9), were developed (24). The IGDS-SF9 comprises nine items based on the DSM-5 IGD criteria and presents several advantages. Its nine items address all nine DSM-5 diagnostic criteria (24), for example, and studies have indicated that it is a robust one-dimensional scale with excellent psychometric properties (25, 26). It is thus attractive for researchers and clinical practitioners to use.

Consequently, IGDS-SF9 has been adapted for use in many cultures. The scale has been validated in Hong-Kong (27), Taiwan (28), Albania, U.K., USA, Italy (29), Iran (30), Poland (31), Germany (32), Spain (33), Korea (34), Malaysia (35), Slovenia (36), and Turkey (37).

Validation of IGDS-SF9 in an Arabic context would contribute to its cross-cultural validation, given the importance of considering cross-cultural perspectives (38). To date, only one validation of an Arabic version of the scale has been carried out (39); however, the study presented several limitations. This research did not address factor structure, measurement invariance, concurrent, convergent, and criterion validity or item theory analysis. Therefore, this study aims to enhance the literature and practice by developing and then investigating the psychometric properties of an IGDS-SF9 Arabic version in a study using a Saudi Arabian student sample.

## 2. Methods

### 2.1. Study design and sample

To involve the maximum number and widest diversity of participants, this quantitative research employed convenience sampling method. This is study was conducted among students. The survey link was sent to participants online via email, Facebook, WhatsApp, and Twitter to inform them of the study’s purpose and intended outcome. Students provided consent before beginning the survey. Students who played videogames in the last 12 months were included in this study. Around 537 respondents returned the survey. Only 410 participants did so with all the data completed and had played videogames in the last 12 months, so they became the study’s sample; their mean age was 17.6 with a standard deviation of 1.56. Around 45.6% were females, 61.5% were high school students and 38.5% were university students. 17% of the participants were from families earning less than 3,000 RS monthly, 17.3% from families earning between 3,000 and 5,000 SR, 7.3% from families earning between 5,000 and 7,000 SR, 8% from families earning between 7,000 and 9,000 SR, 13.2% from families earning between 9,000 and 11,000 SR, and 37% from families earning more than 11,000 SR. Around 63.4% had an active lifestyle and 36.6% had a sedentary one. A total of 72 respondents agreed to be contacted again 3 weeks later for a retest. The mean age for this latter sample was 17.45 with a standard deviation of 1.53, and comprised 37.5% females. The resultant data were utilized for checking the IGDS-SF9’s test–retest reliability.

### 2.2. Measures

The questionnaire sent to respondents comprised the Arabic version of the Internet Gaming Disorder Scale, short form (24), the Smartphone Addiction Scale (SAS) (40), the Smartphone Application-Based Addiction Scale (SABAS) (41), and the Hospital Anxiety and Depression Scale (HADS) (42), and also a range of demographic questions.

#### 2.2.1. The Internet Gaming Disorder Scale, short form

The original version of IGDS-SF9 (24) is a brief measure with nine items based on the nine diagnostic criteria suggested by the DSM-5 (23). Each item is presented in the form of a question such as, *Do you feel more irritability, anxiety, or even sadness when you try to either reduce or stop your gaming activity?*; *Do you feel the need to spend increasing amounts of time engaged in gaming in order to achieve satisfaction or pleasure?*; *Have you lost interest in previous hobbies and other entertainment activities as a result of your engagement with the game?*; and *Have you continued your gaming activity despite knowing it was causing problems between you and other people?* Items were scored on a 5-point Likert scale, with responses ranging from 1 (rarely) to 5 (very often). The scale’s total scores extend from 9 to 45, with pathological gaming related to higher scores.

#### 2.2.2. The hospital anxiety and depression scale

The HADS (42) is a 14-item scale, with seven items to measure anxiety and seven to measure depression. Items include statements such as *I feel tense or “wound up”*, *I get a sort of frightened feeling as if something awful is about to happen*, *I feel cheerful*, and *I feel as if I am slowed down*, and are scored on a Likert scale ranging between 0 and 3 (representing the most negative response for each statement). Thus, the maximum for each of the two subscales is a score of 21. When Terkawi et al. (43) validated the HADS in Saudi Arabia, it also had sound psychometric properties (43). In this present paper, the HADS had good internal consistency reliability coefficients (Cronbach’s alpha = 0.88 for anxiety and 0.72 for depression) and was used to assess the concurrent validity of IGDS-SF9.

#### 2.2.3. The smartphone addiction scale

SAS is a 33-item scale that measures six factors (40), which include daily-life disturbance (5 items: items 1 through 5), positive anticipation (8 items: items 7 through 14), withdrawal (5 items: items 15 through 19), cyberspace-oriented relationships (7 items: 20 through 26), overuse (4 items: items 27 through 30), and tolerance (4 items: item 6 and items 31 through 33). A six-point Likert scale, from 1 (strongly disagree) to 6 (strongly agree), is used for items such as *I will not be able to stand not having a smartphone*, *The people around me tell me that I use my smartphone too much*, and *I feel impatient and fretful when I am not holding my smartphone*. The total score extends from 33 to 198, with higher scores indicating pathological smartphone usage. El Sayed El Keshky et al. (44) translated this scale and validated it in Arabic (44) and it is this version that was used in our study. In this paper, the SAS had good internal consistency reliability (Cronbach’s alpha = 0.73 for daily disturbance, 0.79 for positive anticipation, 0.75 for withdrawal, 0.78 for a cyberspace-oriented relationship, 0.72 for overuse, and 0.79 for tolerance). This scale was used to assess the convergent validity of IGDS-SF9.

#### 2.2.4. The smartphone application-based addiction scale

SABAS is a short measure with items such as My smartphone is the most important thing in my life; I try to cut the time I use my smartphone, I manage to do so for a while, but then I end up using it as much or more than before; Conflicts have arisen between me and my family (or friends) because of my smartphone use; and If I cannot use or access my smartphone when I feel like, I feel sad, moody, or irritable (41). Originally developed in Hungarian, the English version of this scale was validated. The scale’s six items are rated on a 6-point Likert scale from 1 (strongly disagree) to 6 (strongly agree); thus, total scores extend from 6 to 36, with smartphone addiction signified by high scores. Csibi et al. (41) reported sound internal consistency reliability. In this study, SABAS with an internal consistency of 0.72 was used to assess SAS’s convergent validity.

### 2.3. Translation process and pilot study

The process of translating IGDS-SF9 was conducted according to the five-stage recommendation of Beaton et al. (45). In the first stage, two bilingual translators translated the scale from English to Arabic independently. The second stage consisted of a synthesis of the original version and both translators’ versions. Stage 3 was back translation, in which a bilingual translator translated the Arabic synthesized version back to English to check its validity. Stage 4 entailed experts checking for cross-cultural equivalence of the English and Arabic versions. Stage 5 consisted of a pilot study, where the final translation was sent to 65 participants. These participants completed the questionnaire and were interviewed about the clarity and meaning of each item. The main study utilized this final verified version.

### 2.4. Statistical analysis

RStudio statistical software (46) was used for data analysis. The exploratory factor analysis (EFA) and reliability analysis employed the “psych” package in RStudio (47). The confirmatory factor analysis (CFA) was performed using the “lavaan” package (48). The plotting of the CFA model was facilitated by the “lavaanPlot” package (49). Model fit was assessed with the Comparative Fit Index (CFI), Tucker-Lewis Index (TLI), Root Mean Square Error of Approximation (RMSEA), and Standardized Root Square Mean Residual (SRMR) methods (50). According to Hu and Bentler (50), values >0.90 for CFI and TLI and values <0.08 for RMSEA and SRMR signify a sound model fit. Measurement invariance across gender was also investigated following the checklist suggested by Van de Schoot et al. (51) using multigroup CFA. Finally, item response theory was investigated with the polytomous graded response model (52) because the items were polytomous. Its plotting was performed using the “ltm” package (53) in RStudio.

### 2.5. Ethics

All procedures performed in this study were in accordance with the ethical standards of the responsible institutional and national committees on human experimentation and with the Helsinki Declaration. The study was approved by the institutional review board of the Deanship of Scientific Research (DSR) of King Abdulaziz University in Saudi Arabia, number 125-524-D1435. All subjects were informed about the study, and all provided informed consent.

## 3. Results

### 3.1. Exploratory factor analysis

**Figure 1:**
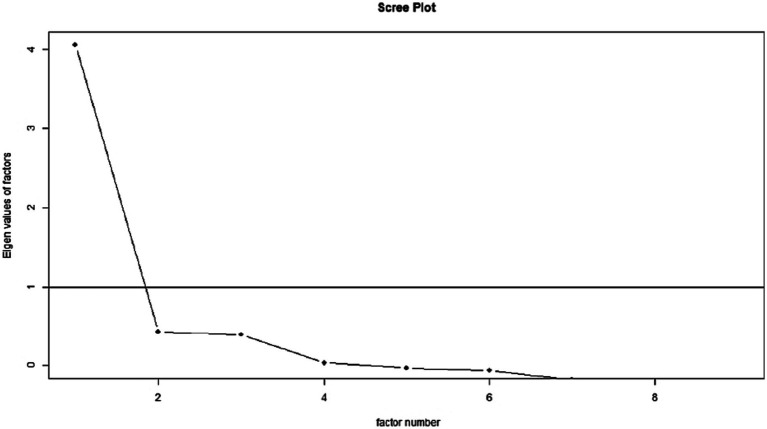
Scree plot of the EFA.

EFA was performed with the maximum likelihood extracting method and the varimax rotation method. The factor adequacy was measured by the Kaiser-Mayer-Olkin (KMO) test, and the sampling adequacy was 0.86. Bartlett’s test of sphericity was significant (Bartlett’s K-squared = 448.19, *df* = 8, *p*-value < 0.001). The factor loadings for each item extended from 0.35 to 0.70. This EFA exhibited a good model fit (χ2 = 123.42, *df* = 27, *p* < 0.001, RMSA = 0.05, TLI = 0.95, RMSEA = 0.04). The scree plot suggested a one-factor model, as in the original study of Pontes and Griffiths (24) (see Figure 1). The EFA results indicated a structure that explained 45% of the variance. The EFA loading for each item appears in Table 1.

**Table 1:** EFA and CFA factor loadings.

	EFA loading	CFA loading
Item 1	0.41	0.50
Item 2	0.35	0.45
Item 3	0.57	0.48
Item 4	0.63	0.35
Item 5	0.54	0.49
Item 6	0.39	0.59
Item 7	0.70	0.64
Item 8	0.49	0.56
Item 9	0.54	0.37

### 3.2. Confirmatory factor analysis of IGDS-SF9

**Figure 2:**
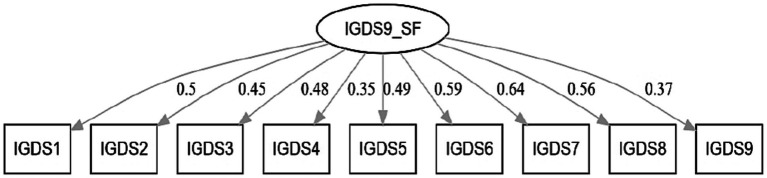
CFA plot for IGDS-SF9.

CFA was performed to check the construct validity and unidimensionality of IGDS-SF9. The CFA showed the following results: χ2 = 165.23, *df* = 31, *p* < 0.001, CFI = 0.95, TLI = 0.93, RMSEA = 0.04, SRMR = 0.03. The CFA factor loadings extended from 0.35 to 0.64. Table 1 displays the CFA loadings for all items, and Figure 2 plots the CFA model.

Multigroup CFA was also carried out to investigate the measurement invariance of IGDS-SF9 across gender. The steps followed were those suggested by Van de Schoot et al. (51), including configural invariance, metric invariance, scalar invariance, and strict invariance. The configural invariance indicated good model fit (χ2 = 139.45, *df* = 26, *p* < 0.001, CFI = 0.94, TLI = 0.93, RMSEA = 0.05, SRMR = 0.04). In the metric invariance analysis, factor loadings were set to be equal across gender and this model exhibited good fit indices (χ2 = 148.75, *df* = 29, *p* < 0.001, CFI = 0.93, TLI = 0.92, RMSEA = 0.05, SRMR = 0.04). The differences in CFI, TLI, SRMR, and RMSEA between the configural and metric model were less than 0.01, which supports metric invariance. In the scalar invariance analysis, the intercepts were added to the metric model and the model showed good model fit (χ2 = 157.92, *df* = 31, *p* < 0.001, CFI = 0.95, TLI = 0.94, RMSEA = 0.05, SRMR = 0.04). The differences in CFI, TLI, SRMR, and RMSEA between the metric invariance and scalar invariance models were less than 0.01, thus supporting scalar invariance. In the strict invariance model, along with the factor loadings and intercepts, residuals were allowed to be equal across gender and the model exhibited good model fit (χ2 = 161.23, *df* = 33, *p* < 0.001, CFI = 0.94, TLI = 0.93, RMSEA = 0.05, SRMR = 0.06). The differences in CFI, TLI, SRMR, and RMSEA between scalar and strict invariance were less than 0.01, and thus supported full uniqueness measurement invariance.

### 3.3. Item response theory

**Figure 3:**
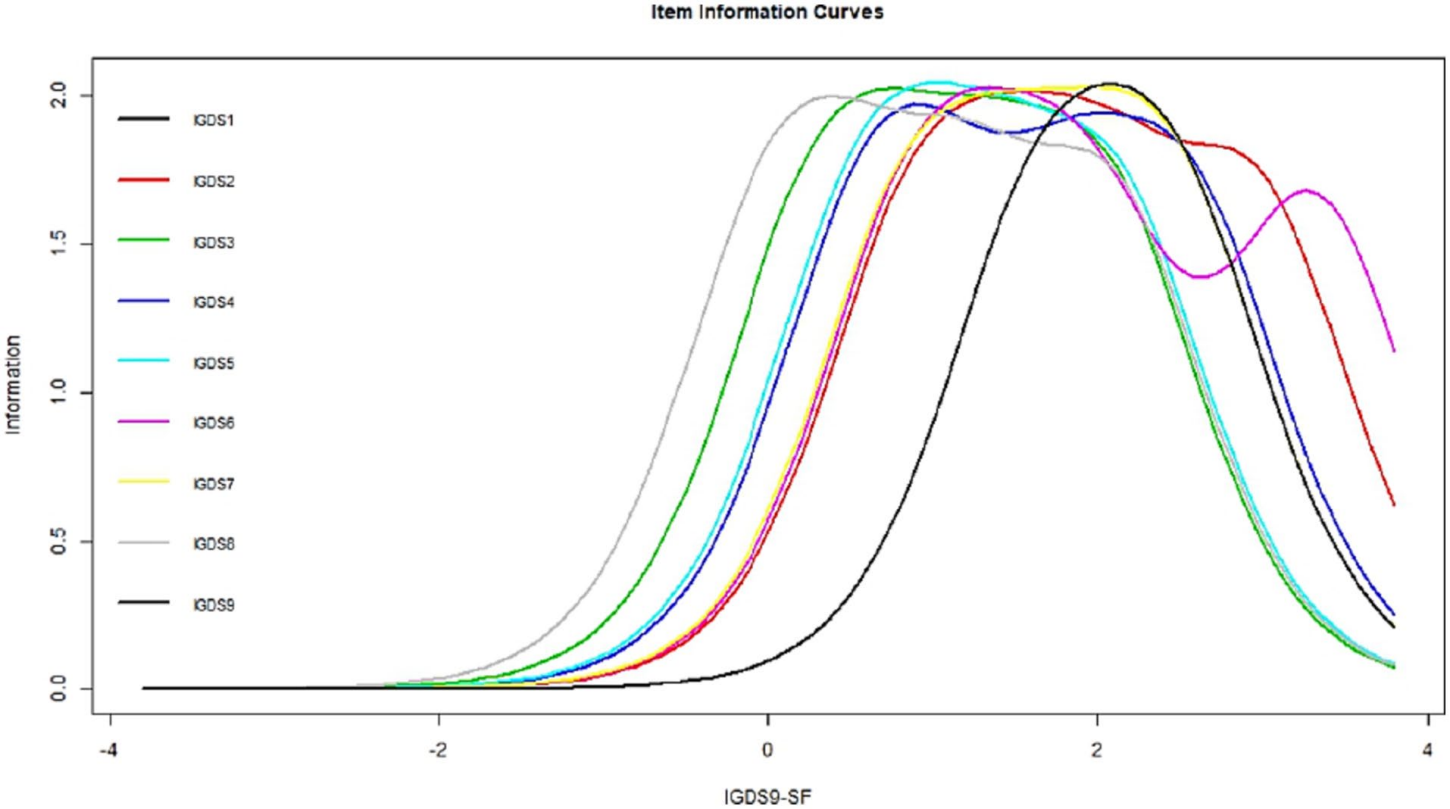
Item response theory for IGDS-SF9.

Item response theory provides an understanding of how effectively the latent dimension for each item is measured at different levels of the construct. The results are plotted in Figure 3; all the items follow the same pattern and provide more information at higher levels of the construct levels.

### 3.4. Convergent validity

The convergent validity was established by using correlations with the SAS and the SABAS. All the correlations were significant at *p* < 0.001 and extended from 0.35 to 0.41, indicating moderate convergent validity of IGDS-SF9.

### 3.5. Concurrent validity

The concurrent validity was established by using correlations with the HADS and were all significant at *p* < 0.001. Its correlations were significant at *p* < 0.001 and extended from 0.27 to 0.33, indicating moderate concurrent validity of IGDS-SF9.

### 3.6. Criterion validity

The criterion validity of IGDS-SF9 was established with correlations with the weekly time spent playing videogames and the average hours per gaming session. Extending from 0.55 to 0.59, all correlations were significant at *p* < 0.001 and indicate good Criterion validity. Table 2 summarizes them.

**Table 2:** Criterion validity.

	IGDS-SF9
Weekly time spent gaming	0.55***
Average per gaming session	0.59***

***p < 0.001.

### 3.7. Reliability analysis

Table 3 summarizes the mean scores and standard deviations for IGDS-SF9, its items and Cronbach’s alpha and test–retest coefficients. The mean score for IGDS-SF9 was 14.16, with a standard deviation of 6.08, which shows, on average, there is a moderate prevalence of IGD in the sample. The internal consistency reliability coefficient was 0.87, and the test–retest reliability coefficient was 0.84, which indicates good internal consistency reliability and temporal stability of the scale.

**Table 3:** Mean, SD, internal consistency reliability, and test–retest reliability.

	Mean	SD	Cronbach’s alpha	Test–retest
IGDS-SF9	14.16	6.08	0.87	0.84
Item 1	1.43	0.87		
Item 2	1.38	0.79		
Item 3	1.88	1.19		
Item 4	1.61	0.98		
Item 5	1.77	1.19		
Item 6	1.43	0.87		
Item 7	1.42	0.85		
Item 8	2.07	1.23		
Item 9	1.13	0.49		

## 4. Discussion

For decades, excessive use of online gaming has attracted the attention of academics and clinicians. IGD is recognized as a disorder by the American Psychological Association, which established criteria for its diagnosis. Consequently, several measures were developed, but IGDS-SF9 received more attention due to its brief, concise, and psychometrically sound nature. Given that it was found that interventions can be successful in preventing the development and progression of online gaming symptoms in problematic population and normal population (54, 55), it is important to validate measures that can be used for diagnosis, such as IGDS-SF9.This scale has been translated into multiple languages. An Arabic version has been validated previously but without investigating confirmatory factor analysis, measurement invariance, and item response theory. Thus, this study aimed to develop a new Arabic version for IGDS-SF9 and investigate its psychometric properties.

EFA for the Arabic IGDS-SF9 showed a unidimensional structure, similar to the original research by Pontes and Griffiths (24). The CFA exhibited good fit indices and adequate factor loadings. The significant correlations of the Arabic IGDS-SF9 with the SAS, SABAS, HADS, weekly time spent playing videogames, and average hours per gaming session confirmed the scale’s convergent, concurrent, and criterion validity. Convergent validity, concurrent validity, and criterion validity were also reported in previous studies with alternative versions of IGDS-SF9 (34, 56–58). Its internal consistency reliability and measurement invariance across gender were also confirmed, and the item response theory showed that the items were monotonic. Previous research also reported good reliability of other IGDS-SF9 versions (9, 25, 34, 36, 57–59).

The EFA results showed that the data were suitable for factorability and that the sampling adequacy was also good. The EFA factor loadings were also adequate. The scree plot revealed the scale’s unidimensional structure, which mirrors earlier research (59). CFA was carried out for the unidimensional IGDS-SF9, resulting in satisfactory fit indices and adequate factor loadings comparable to those from earlier validation studies (35, 36, 59). Cross-gender measurement invariance was also established following Van de Schoot et al. (51) and mirrored earlier research reporting the same (9) even in different countries (25, 26). Item response theory indicated that the items were monotonic and provided more detail at the construct’s high levels. Another study by Schivinski et al. (31) validated IGDS-SF9 using item response theory and reported similar results. With these evidence, this study confirms the cross-cultural validity of IGDS-SF9 that was reported in previous studies (26). As such, this study contributes to the literature with new evidence of psychometric properties of the scale from another cultural context.

These psychometric properties signify the validity and reliability of the Arabic version of IGDS-SF9; it is eminently usable in Arabic countries. Koronczai et al. (60) suggested that an appropriate scale would be short and comprehensive, as well as having reliability and validity for various types of data collection and age groups, and cross-cultural research; and validated with clinical samples. Studies have shown that the DSM-5 criteria for IGD fulfill these requirements (61, 62). As the IGDS-SF9 was developed according to these DSM-5 criteria, its scale is short, valid, reliable, and psychometrically sound in different cultures, and is an excellent candidate for ease of use in research and practice.

### 4.1. Implications of the study

This study’s results present implications for theory and practice. This study showed that the IGDS-SF9 was psychometrically sound and was a good indicator across both gender categories in a different cultural context, which adds new evidence to the literature. For researchers and clinicians, iIt is important that researchers investigate the prevalence of IGD at a regional or country level, and with the Arabic version of IGDS-SF9, that is now possible in Arabic contexts. It is important that clinicians diagnose IGD so they can provide therapeutic interventions, which is now possible as well in an Arabic context.

### 4.2. Limitations

Some limitations of this study must be acknowledged. Firstly, this study used a cross-sectional design which is not ideal for generalizability of findings. Secondly, it adopted a convenience sampling method, random sampling would be preferable in future research. Thirdly, it relied upon subjective measures, objective measures should be also used in future research to bring additional insights. Fourthly, this study was conduced among students, interpretation of findings should be done baring this in mind.

## 5. Conclusion

This research developed and investigated the psychometric properties of an Arabic version of IGDS-SF9. The results signify that it is valid and reliable for Arabic-speaking countries. The EFA results were analogous to those of the Pontes and Griffiths (24) original study; the CFA results indicated good model fit and factor loading. The scale was gender invariant; item response theory provided information about the items; concurrent, convergent, and criterion validity were established, and there was sufficient internal consistency reliability and test–retest reliability.

## Data availability statement

The original contributions presented in the study are included in the article/supplementary material, further inquiries can be directed to the corresponding author.

## Ethics statement

The studies involving humans were approved by the institutional review board of the Deanship of Scientific Research (DSR) of King Abdulaziz University in Saudi Arabia. The studies were conducted in accordance with the local legislation and institutional requirements. The participants provided their written informed consent to participate in this study. Written informed consent was obtained from the individual(s) for the publication of any potentially identifiable images or data included in this article.

## Author contributions

ME contributed to the definition of research objectives, model and hypotheses. ME and TA participated in data analysis plan, writing - original draft, and approval of the final manuscript. ME and TA contributed to the provision of materials (i.e., questionnaires), participated in data collection, review and editing. All authors and have read and approved the final version of the manuscript.
