# Corrigendum: Factor structure and psychometric properties of an Arabic version of the Internet Gaming Disorder Scale, short form (IGDS-SF9)

**DOI:** 10.3389/fpubh.2024.1493887

**Published:** 2024-12-30

**Authors:** Mogeda El Sayed El Keshky, Tmader Alballa

**Affiliations:** ^1^Department of Psychology, Faculty of Arts and Humanities, King Abdulaziz University, Jeddah, Saudi Arabia; ^2^Department of Mathematics, College of Sciences, Princess Nourah bint Abdulrahman University, Riyadh, Saudi Arabia

**Keywords:** internet gaming disorder, Internet Gaming Disorder Scale (IGDS-SF9), psychometric properties, scale validation, factor structure, Saudi Arabia

In the published article, the Funding statement was missing. The correct Funding statement appears below.

